# Evaluation of *SORD* mutations as a novel cause of Charcot‐Marie‐Tooth disease

**DOI:** 10.1002/acn3.51268

**Published:** 2020-12-12

**Authors:** Ru‐Ying Yuan, Zi‐Ling Ye, Xiao‐Rong Zhang, Liu‐Qing Xu, Jin He

**Affiliations:** ^1^ Department of Neurology and Institute of Neurology First Affiliated Hospital Institute of Neuroscience Fujian Key Laboratory of Molecular Neurology Fujian Medical University Fuzhou 350005 China

## Abstract

Biallelic mutations in the sorbitol dehydrogenase (SORD) encoding gene were recently identified as a common genetic cause in autosomal‐recessive CMT patients. Here, we investigated the clinical, genetic, and electrophysiological characteristics of three CMT patients with biallelic SORD mutations from a Chinese cohort. Two patients harbored c.757delG (p.A253Qfs*27) homozygous mutations, and one patient carried both c.757delG (p.A253Qfs*27) and c.625C>T (p.R209X) compound heterozygous mutations. Interestingly, the two patients homozygous for the c.757delG mutation exhibited positive responses for pinprick test. In conclusion, we confirmed *SORD* mutations as causative for CMT and further expanded the mutational and phenotypic spectrum of *SORD*‐related CMT.

## Introduction

Charcot–Marie–Tooth (CMT) disease is the most common inherited peripheral neuropathy, with a prevalence of 1 in 2500 individuals.^1^ CMT is most commonly characterized by distal wasting, weakness, and sensory loss that starts in the lower limbs and progresses slowly in a length‐dependent manner.^2^ Based on neurophysiological symptoms, CMT is classified into two main types: demyelinating CMT1 and axonal CMT2, using upper limb motor conduction velocities (MCVs) to define type 1 as having MCVs < 38 m/s and type 2 with MCVs > 38 m/s.Distal hereditary motor neuropathy (dHMN) has a broad clinical and genetic overlap with axonal CMT. Distal hereditary motor neuropathy (dHMN) represents a form of CMT2 in which the burden of disease falls predominantly or exclusively on motor nerves. This classification remains the cornerstone of modern diagnosis.^3^ To date, more than 100 causative genes for this disease have been reported worldwide. Thus, for a large proportion of CMT patients the genetic basis remains unexplained.^1^, ^4^ Very recently, biallelic mutations in the sorbitol dehydrogenase (*SORD)* gene were identified as a novel and common genetic cause for autosomal‐recessive CMT. The discovery of the gene may resolve the genetic diagnosis of a significant proportion of CMT patients.^5^

In order to investigate the clinical, electrophysiological, and genetic features of CMT patients with mutations in the *SORD* gene, here, in this work, we evaluate detailed clinical and electromyographic data, combined with mutation analysis of the SORD gene, from a CMT patient cohort in China.

## Methods

A total of 215 CMT patients from a registered cohort study on Charcot–Marie–Tooth disease (NCT04010188) had genomic DNA extracted from peripheral blood using standard protocols. Samples were first screened for 17p11.2 copy number variation, *GJB1*, and *MFN2* mutations, which are the most commonly reported causative mutations for CMT. We then used whole exome sequencing (WES) to screen for other CMT‐related mutations. All nine exons of the SORD (NM_003104) gene were amplified by PCR. All candidate mutations were validated using Sanger sequencing and classified according to the American College of Medical Genetics and Genomics (ACMG) standards and guidelines.

Serum sorbitol dehydrogenase was quantified using a sorbitol dehydrogenase ELISA kit (Human sorbitol dehydrogenase ELISA kit, JL12961, Jianglaibio, Shanghai).

The study was approved by the Ethics Committee of the First Affiliated Hospital of Fujian Medical University. Written informed consent was obtained from all participants.

## Case Reports

Patient 1, a 24‐year‐old male college student, visited the clinic with complaint of progressive muscle atrophy and weakness of the distal lower limbs over 7 years. He also often presented uncontrollable muscle jumping in limbs, especially in thenar muscles. He had gait abnormality and slight decline of fine motor function in upper limbs. He presented with atrophy above ankle to lower half of the leg and under the wrist joints. On clinical examination, the strength of dorsal flexor muscles in left foot disappeared. The myodynamia of left flexor digitorum and right distal lower limbs achieved grade 4. Achilles reflex as well as Babinski's sign were negative. Nerve conduction velocity (NCV) showed axonal form and CMTNS score was graded 9.^6^ In the follow‐up,intriguingly, this patient also exhibited the positive dermographism sign,just like patient 2 .

Patient 2, a 35‐year‐old female, had the longest course among three patients with a history of 19 years. Symptom first appeared as weakness and atrophy of lower limbs. She found migratory muscle abnormal twitching and occasional pain in calves during the course. Without intellectual development retardation, this patient had worse athletic performance compared with her peers. Physical examination showed the distal lower limbs atrophy and weakness(4/5), and deformed feet. Gloves hypoesthesia appeared in hands to wrist joints and feet to ankle joints. Nerve conduction velocity (NCV) showed axonal form. The patient mentioned in the medical history that the skin is prone to wheal and flare response, when it is physically irritated. Notably, dermographism we performed on him was positive.

Patient 3, a 22‐year‐old young man, began to notice the atrophy of lower legs when he was 15‐years‐old. Then he found that his lower limbs became weaker gradually. Luckily, to date no other complications were observed. Compared to the other two patients, the strength of his lower extremities was the weakest with only grade 2. Unfortunately, we only completed the neuroelectrophysiological examination for the lower limbs, and thus we could not accurately evaluate the CMTNS Score.

## Results

**Figure 1:**
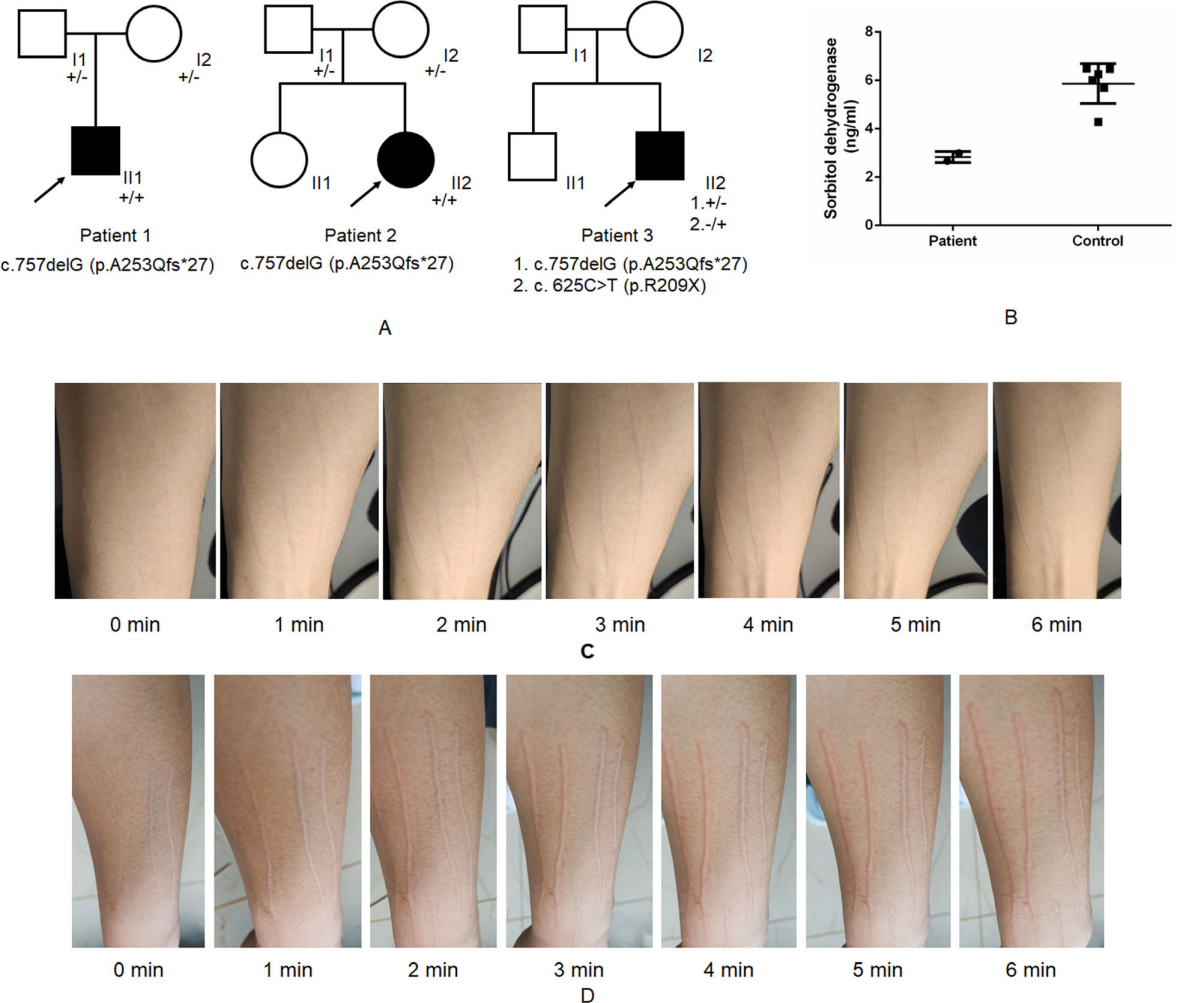
Family segregation analysis, sorbitol dehydrogenase analysis, and pinprick tests of patients with *SORD* mutations. (A) The pedigree and familial segregation of the three patients. (B) Sorbitol dehydrogenase analysis using ELISA for patients 1 and 2, compared with six normal controls. (C) Pinprick tests of patient 1. (D) Pinprick tests of patient 2

Three patients in the cohort were found to harbor *SORD* mutations (Figure 1). Two patients carried *SORD* (NM_003104): c.757delG (p.A253Qfs*27) homozygous mutations, and one patient harbored *SORD* (NM_003104): c.757delG (p.A253Qfs*27) and c.625C>T (p.R209X) compound heterozygous mutations (Figure 1 and Figure S1). The c.757delG mutation was previously reported in CMT patients, but the c.625C>T (p.R209X) mutation has not yet been identified in CMT patients. The allele carrier frequency of c.625C>T (p.R209X) in the control population was 0.00002305, based on an allelic count of 3 out of 130176 genomes in the gnomAD v2 database. This allele has thus far not been found in east Asian populations. Because the parents of patient 3 rejected genetic testing, we were unable to obtain data on familial co‐segregation for this subject. According to ACMG standards and guidelines, the c.625C>T (p.R209X) mutation is currently classified as a variant of very strong pathogenicity (PVS1, PM2, PP3).

The clinical manifestations of the three patients were very similar (Table S1). The age at onset ranged from 14 to 16 years old, and the onset symptom started from lower limbs. Neurological examinations revealed obvious weakness and atrophy of both distal lower limbs, and no evident weakness or atrophy of upper limbs. The deep tendon reflexes were decreased or absent in distal lower limbs, but normal in upper limbs. Babinski’s sign was absent bilaterally. No sensory disturbance or cognitive impairment were observed. Nerve conduction studies revealed decreased compound muscle action potential (cMAP) in the nerves of lower limbs, with normal motor nerve conduction velocity (MNCV). Both cMAP and MNCV of upper limbs were normal, as was sensory nerve conduction. Interestingly, dermographism of patient 1 and patient 2 were positive (Figure 1). We also tested serum sorbitol dehydrogenase levels in patients 1 and 2 and found that their serum levels of sorbitol dehydrogenase were lower than that of healthy control subjects (Figure 1).

## Discussion

In our study, the *SORD* mutation frequency was 1.39% (3/215) among patients with CMT, and 7.5% (3/40) among patients with CMT2, which was lower than the mutation frequency of *MFN2* (37.5%, 15/40).^7^ Mutations in the *SORD* gene represent the second most common genetic basis of CMT2 in our cohort.

Here, we also summarized the clinical characteristics of patients with *SORD* mutations. All three patients present with muscle weakness and atrophy of distal lower limbs but do not present with symptoms in upper limbs. The neurology examination and nerve conduction studies of sensory nerves were normal. It is worth noting that the two patients with c.757delG (p.A253Qfs*27) homozygous mutations exhibited positive signs in dermographism. There's literature that says sorbitol concentrations influences IgE‐mediated histamine release from human basophilsd and can stimulates part of immune pathway through polyol pathway. Whether the two are involved in the occurrence of this phenomenon still needs further study and discussion.^8^, ^9^One study showed that a persistent flare response in human skin depends on the presence of the excitation of mechano‐insensitive C‐fibers on the axon.^10^ In a previous study, J.W. Ralph reported a case with cryptogenic distal axonal polyneuropathy. When the patient did dermographism, no skin scratches presenting in the distal area of the upper limb. J.W. Ralph. think that the absence of dermographism in distal regionsis best explained by cutaneous denervation in those regions, where the C‐fibers responsible for the axon reflex flare are missing.^11^ This is not the case in our patients. However, long‐term follow‐up observation is needed to determine whether this phenomenon will occur as the disease progresses.

The *SORD* gene encodes sorbitol dehydrogenase, which catalyzes the reversible NAD(+)‐dependent oxidation of sorbitol into fructose. Mutations in the *SORD* gene result in decreased levels of sorbitol dehydrogenase, which consequently affects sorbitol accumulation. In our study, we also found that the c.757delG homozygous mutation led to a decrease in serum levels of sorbitol dehydrogenase. We confirmed that the *SORD* mutation led to CMT2, but the mechanism by which SORD deficiency incurs axonal damage remains unclear. Previous research has shown that the accumulation of sorbitol in the sciatic nerve induced neuropathy in a diabetic mouse model.^12^ More recently, the effects of SORD deficiency on neurodegeneration were investigated in an *Sodh2^MB01265/MB01265^* drosophila model.^5^ However, more evidence is necessary to definitively elucidate the neuropathic mechanisms associated with *SORD* mutation. Furthermore, the allele carrier frequency for the c.757delG variant indicates that it is common in population data, and that mutations in *SORD* represent the second most common cause of autosomal recessive CMT2 in our cohort. These findings suggest that future strategies for therapeutic intervention in CMT patients, such as base editing gene therapy, may be able to directly target *SORD* mutations, like the c.757delG variant, to reverse the symptoms associated with loss of sorbitol dehydrogenase function.

## Author Contributions

J.H., RY.Y., ZL.Y., XR.Z., and LQ.X. collected and analyzed the data. J.H. and RY.Y. drafted the manuscript. J.H. critically revised and gave final approval for publication of the paper.

## Conflict of Interest

This work has been supported by grants 81870902 (N.W.), U190520142 (W.‐J.C.), and 81771230 (W.‐J.C.) from the National Natural Science Foundation of China, grants 2018Y9082 (N.W.) and 2017Y9094 (W.‐J.C.) from the Joint Funds for the Innovation of Science and Technology of Fujian Province, the Key Clinical Specialty Discipline Construction Program of Fujian (N.W.).

## Additional Contributions

We thank the patients for granting permission to publish this information. Written consent for publication was obtained from all participating patients.

## Supporting information


**Figure S1.** Sanger sequencing of the *SORD* mutation in the three patients.

**Figure S2.** Sorbitol analysis using ELISA for patients 1 and 2, compared with six normal controls.

**Table S1.** Clinical manifestations of CMT patients with *SORD* mutations.
