# Supplementary figures and images for: Evaluation of SORD mutations as a novel cause of Charcot‐Marie‐Tooth disease
Source: Ann Clin Transl Neurol. 2020 Dec 12;8(1):266–70. doi: 10.1002/acn3.51268 (PMC7818235; doi:10.1002/acn3.51268)

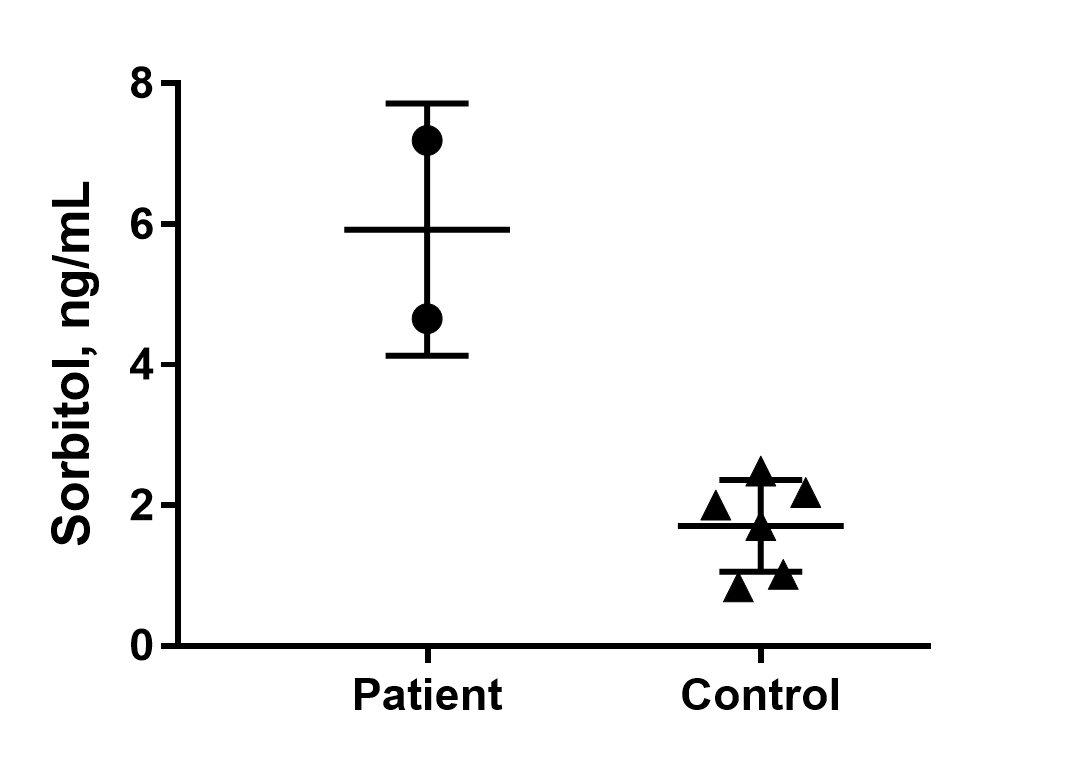

Supplement: Supplementary file 2 — Figure S2. Sorbitol analysis using ELISA for patients 1 and 2, compared with six normal controls. [file ACN3-8-266-s002.tif]
