# Supplementary material for: Evaluation of SORD mutations as a novel cause of Charcot‐Marie‐Tooth disease
Source: Ann Clin Transl Neurol. 2020 Dec 12;8(1):266–70. doi: 10.1002/acn3.51268 (PMC7818235; doi:10.1002/acn3.51268)
Supplement: Supplementary file 3 — Table S1. Clinical manifestations of CMT patients with SORD mutations. [file ACN3-8-266-s003.docx]

Table S1 Clinical manifestations of CMT patients with *SORD* mutations

|  | Patient 1 | Patient 2 | Patient 3 |
| --- | --- | --- | --- |
| Age at onset (years) | 14 | 16 | 15 |
| Gender | male | female | male |
| Clinical subtype | CMT2 | CMT2 | CMT2 |
| Onset symptoms | Muscle weakness and atrophy | Muscle weakness and atrophy | Muscle atrophy |
| Site of onset symptoms | LL | LL | LL |
| Limb weakness UL (MRC) | distal: 5 | distal: 5 | distal: 5 |
| Limb weakness LL (MRC) | distal: 3 | distal: 4 | distal: 2 |
| Pinprick sensation | normal | normal | normal |
| Topesthesia | normal | normal | normal |
| Biceps DTRs | normal | normal | normal |
| Radioperiosteal DTRs | decrease | decrease | normal |
| Knee DTRs | normal | decrease | normal |
| Ankle DTRs | absent | decrease | absent |
| Babinski’s sign | negative | negative | negative |
| Skin-prick test | positive | positive | unknown |
| Age at NCS (years) | 24 | 34 | 21 |
| Median nerve MNCV (m/s) | 57.1 | 58.3 | unknown |
| Median nerve cMAP (mV) | 18.5 | 19.3 | unknown |
| Median nerve SCV (m/s) | 50 | 54.2 | unknown |
| Median nerve SNAP (uV) | 29 | 21 | unknown |
| Peroneal nerve MNCV (m/s) | 41 | 52.7 | 40 |
| peroneal nerve cMAP (mV) | 0.5 | 0.7 | 2.7 |
| Sural nerve SCV (m/s) | 41.7 | 41.4 | 43 |
| Sural nerve SNAP (uV) | 25 | 30 | 37 |
| CMTNS score | 9 | 4 | unknown |

UL: upper limbs, LL: lower limbs, MRC: Medical Research Council scale, DTR: deep tendon reflex, NCS: nerve conduction studies, MNCV: motor nerve conduction velocity, cMAP: compound motor action potential, SCV: sensory conduction velocity, SNAP: sensory nerve action potential.
